# From Valve to Spine and Spleen: Multisystem Complications of *Gemella haemolysans* Prosthetic‐Valve Endocarditis

**DOI:** 10.1155/cric/3705834

**Published:** 2026-11-08

**Authors:** Eric Baghdasaryan, Sari Lada, Rafi Orphali, Ronak Jayswal, Sujay Dutta

**Affiliations:** ^1^ Los Robles Regional Medical Center Thousand Oaks California USA

**Keywords:** discitis, *Gemella haemolysans*, infective endocarditis, prosthetic valve endocarditis, psoas abscess, septic embolism, splenic infarction, TAVR, vertebral osteomyelitis

## Abstract

*Gemella haemolysans* is an uncommon cause of infective endocarditis, yet infection involving a prosthetic valve may be accompanied by extensive extracardiac disease. A 79‐year‐old man with a history of transcatheter aortic valve replacement presented with fever, rigors, dyspnea, severe low back pain, and inability to walk after a fall. Two sets of blood cultures grew *G. haemolysans*. Echocardiography demonstrated a 0.6 × 0.6 cm vegetation at the aortic valve annulus. Lumbar magnetic resonance imaging identified discitis and vertebral osteomyelitis at L4–L5 and L5–S1, an anterior epidural abscess, and moderate‐to‐severe spinal canal stenosis. Abdominal imaging revealed a right psoas abscess and a 6.2‐cm splenic infarction. Antimicrobial therapy was narrowed to intravenous ceftriaxone according to susceptibility testing. Fever resolved and inflammatory markers declined during hospitalization; a 6‐week course was arranged at discharge without valve surgery. This case highlights the association between endocarditis and vertebral infection, the limitations of vegetation size as a measure of extracardiac disease, and the complexity of treatment decisions following transcatheter valve replacement.

## 1. Introduction

Infective endocarditis (IE) remains associated with substantial morbidity and mortality. Although most cases are caused by staphylococci, streptococci, or enterococci, uncommon organisms such as *Gemella haemolysans* are also implicated [1].

*G. haemolysans* is a facultatively anaerobic Gram‐positive coccus found in the normal oral and upper respiratory tract flora. Initially described by Thjøtta and Bøe in 1938 and subsequently assigned to the genus *Gemella* by Berger, the organism was historically difficult to distinguish from streptococci and neisseriae [2, 3]. Improved methods of identification have facilitated recognition of Gemella bacteremia [4].

Although uncommon, *G. haemolysans* endocarditis has been reported in patients with prosthetic valves, and a recent registry confirms that *Gemella* species account for a small fraction of IE episodes [5, 6]. The oropharynx is a possible portal of entry even when no recent dental procedure or overt oral infection is identified.

Vertebral osteomyelitis and discitis are recognized manifestations of hematogenous infection and may complicate IE [7]. We report *G. haemolysans* prosthetic‐valve endocarditis following transcatheter aortic valve replacement (TAVR), accompanied by multilevel vertebral infection, an epidural abscess, a psoas abscess, and splenic infarction. The case raises questions about recognition of concurrent spinal infection, interpretation of a small valvular vegetation, and selection of treatment in patients with TAVR‐associated IE.

## 2. Case Presentation

A 79‐year‐old man was brought to the emergency department after a fall left him unable to walk. During the preceding several days, he had developed progressive dyspnea, fever, rigors, and severe low back pain. His medical history included hypertension, hyperlipidemia, atrial fibrillation, heart failure with preserved ejection fraction, and severe aortic stenosis treated with TAVR in 2023. He reported five falls during the preceding year, including the event that prompted admission. He had undergone no recent dental procedures and knew of no oral infection.

At presentation, his temperature was 38.6°C; he was tachycardic and appeared ill. He was unable to ambulate normally but had no focal neurologic deficits. The white blood cell count was 20 × 10^9^/L, with 92% neutrophils; C‐reactive protein was 121 mg/L, and the erythrocyte sedimentation rate was 77 mm/h. High‐sensitivity troponin was 292 ng/L, and B‐type natriuretic peptide was 321 pg/mL.

Both sets of blood cultures grew *G. haemolysans*, identified by matrix‐assisted laser desorption/ionization time‐of‐flight mass spectrometry (MALDI‐TOF). The isolate was susceptible to the antimicrobial agents tested.

Transthoracic echocardiography revealed a 0.6 × 0.6 cm vegetation at the aortic valve annulus and a left ventricular ejection fraction of 40%–45%. Subsequent transesophageal echocardiography confirmed involvement of the prosthetic valve and better characterized the perivalvular structures (Figures 1 and 2). Neither a perivalvular abscess nor a fistula was identified.

**Figure 1:**
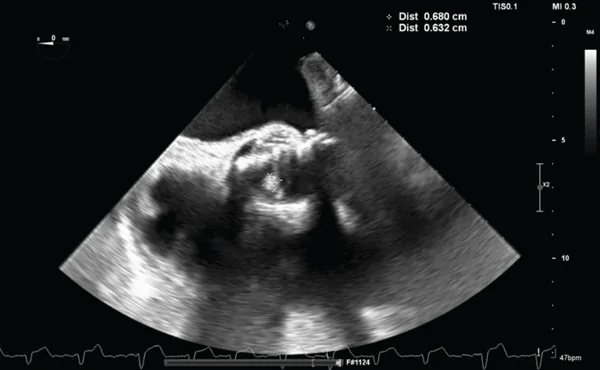
Echocardiographic image showing a 0.6 × 0.6 cm echogenic lesion interpreted as a vegetation at the aortic valve annulus.

**Figure 2:**
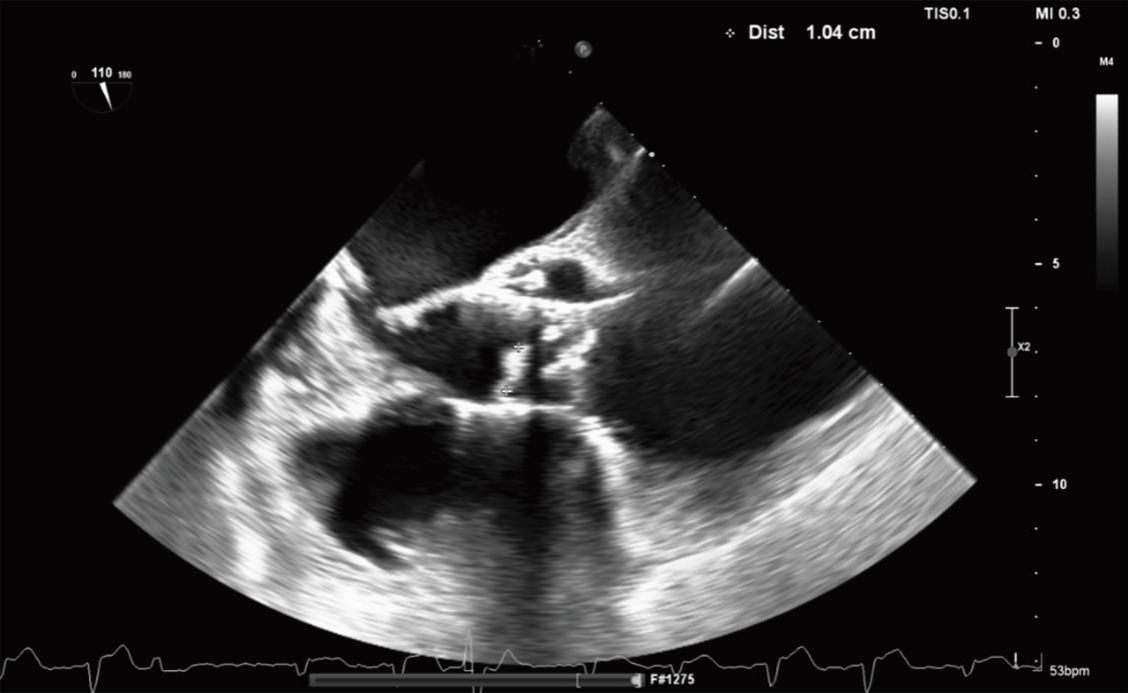
Transesophageal echocardiogram confirming involvement of the aortic valve prosthesis. No perivalvular abscess or fistula was identified.

Abdominal MRI demonstrated a 6.2 cm wedge‐shaped splenic signal abnormality consistent with infarction (Figure 3). Fluid adjacent to the right psoas muscle was interpreted as a psoas abscess. Cross‐sectional imaging of the chest showed no pulmonary emboli or additional infectious foci.

**Figure 3:**
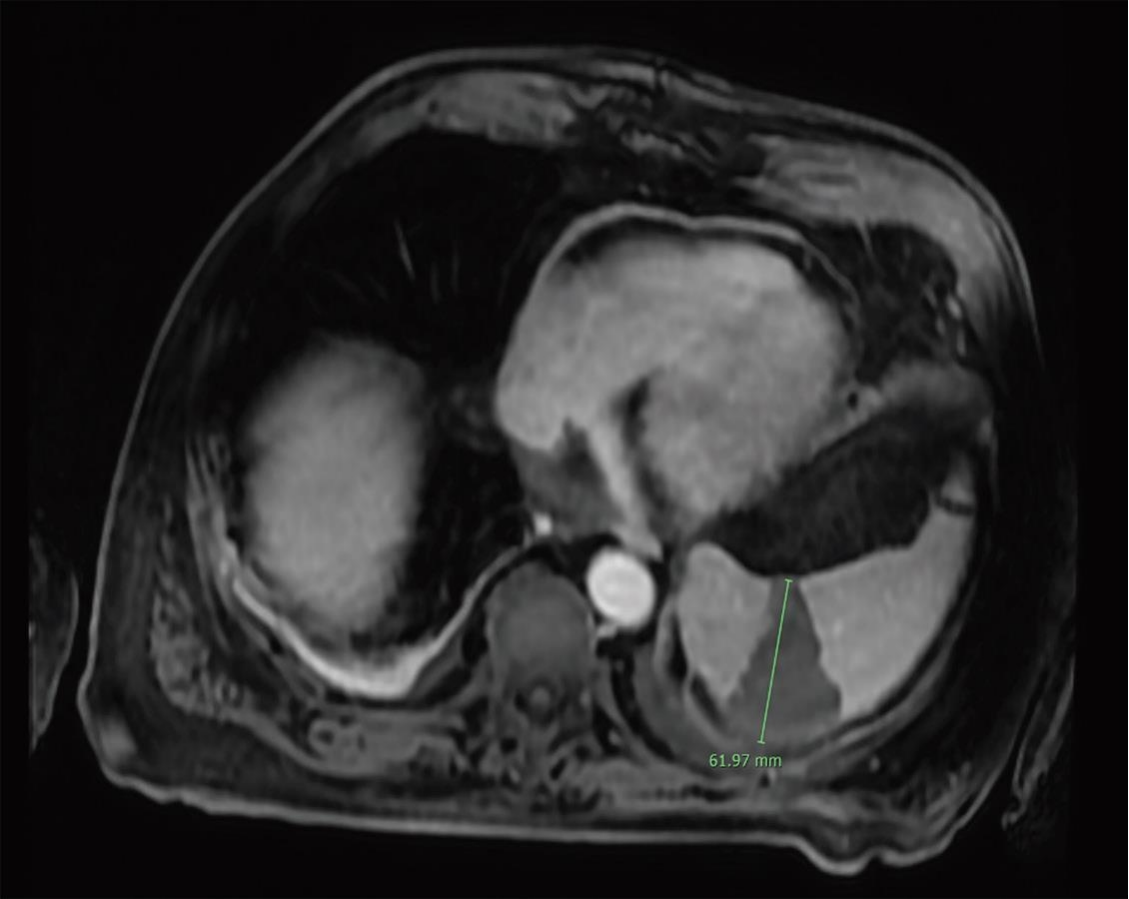
Axial abdominal MRI showing a 6.2 cm wedge‐shaped splenic signal abnormality consistent with infarction. A right psoas collection was also reported on abdominal imaging but is not shown in this panel.

The severity of the back pain and the inability to walk prompted magnetic resonance imaging (MRI) of the lumbar spine. Fluid signal in the L4–L5 and L5–S1 intervertebral discs, together with adjacent vertebral marrow edema, was consistent with discitis and osteomyelitis. Anterior epidural thickening posterior to L5 measured up to 5.5 mm and contributed to moderate‐to‐severe canal stenosis at L5–S1 and moderate stenosis at L4–L5. Moderate bilateral foraminal stenosis was also present at both levels (Figure 4).

**Figure 4:**
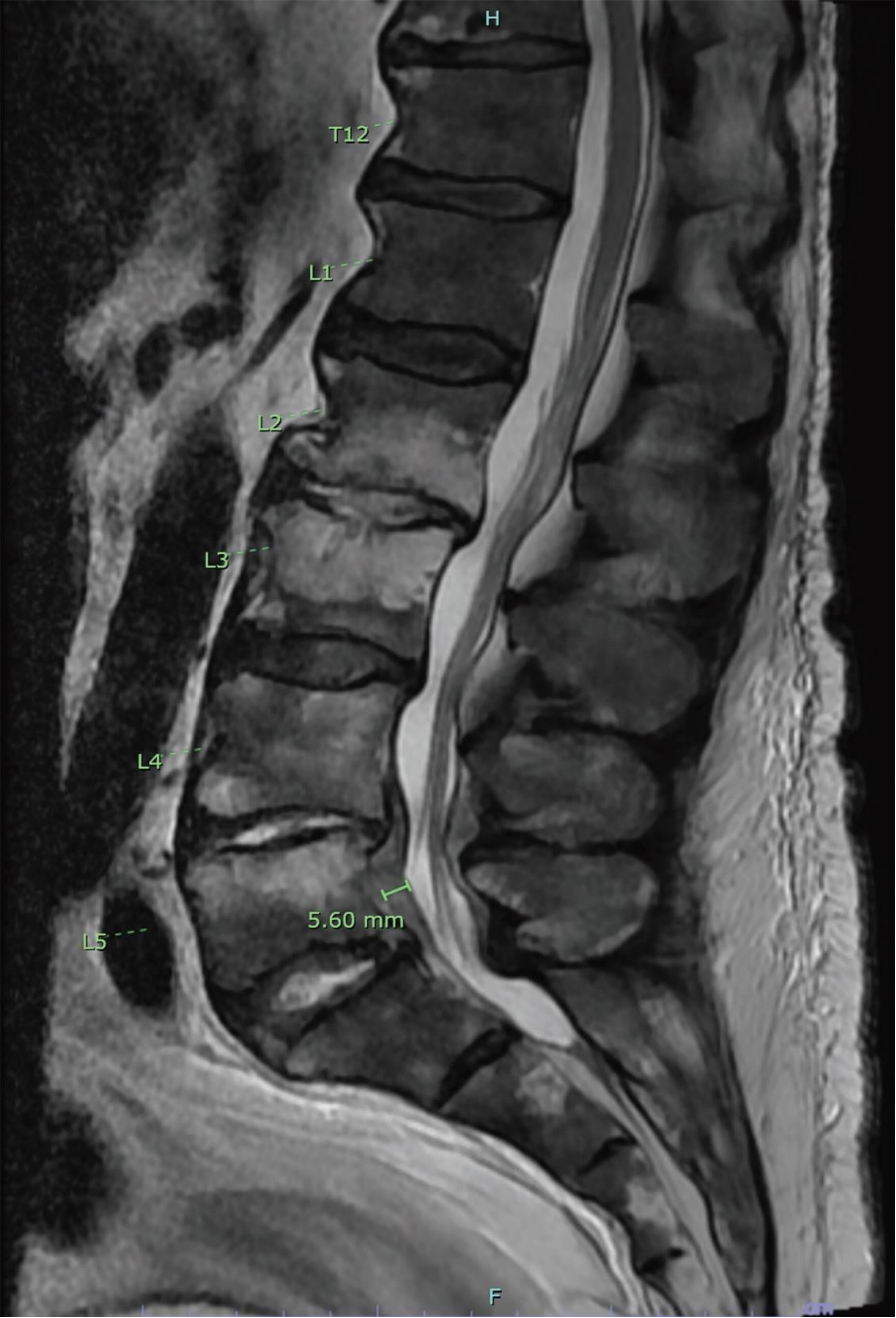
Sagittal lumbar MRI showing L4–L5 and L5–S1 discitis and adjacent vertebral osteomyelitis, with anterior epidural thickening measuring up to 5.5 mm and associated canal stenosis.

The positive blood cultures and echocardiographic vegetation met the 2023 Duke–International Society for Cardiovascular Infectious Diseases criteria for definite IE [8]. Vertebral infection, epidural abscess, a right psoas collection, and splenic infarction demonstrated extensive extracardiac involvement.

Empiric treatment with intravenous vancomycin and ceftriaxone was begun at admission. Once the organism had been identified and susceptibility results were available, vancomycin was discontinued and ceftriaxone continued at 2 g intravenously every 24 h. A peripherally inserted central catheter was placed to allow a planned 6‐week course, in view of the prosthetic valve and concomitant vertebral infection.

The treating team considered the patient a poor operative candidate because of his age, comorbidities, and clinical course. During hospitalization, fever resolved and inflammatory markers declined. He developed no focal neurologic deficits attributable to the epidural abscess and was discharged to complete intravenous treatment with outpatient infectious disease follow‐up.

## 3. Discussion

This case illustrates three clinically important considerations in *G. haemolysans* prosthetic‐valve IE: the association with vertebral osteomyelitis, the limitations of vegetation size in assessing extracardiac complications, and the complex choice between medical and surgical treatment after TAVR. Gemella endocarditis is rare; in a Swedish registry, *Gemella* species accounted for 29 of 7677 IE episodes (0.4%) [6]. Its rarity should not obscure the significance of symptoms that suggest infection beyond the valve.

The first consideration is the bidirectional diagnostic relationship between IE and vertebral osteomyelitis. In a hospital‐based study, concurrent IE was identified in 28 of 91 patients (30.8%) with pyogenic vertebral osteomyelitis; in six patients, echocardiography established IE despite the absence of clinical cardiac findings [9]. Conversely, a contemporary cohort of 633 patients with IE found vertebral osteomyelitis in 73 (11.5%) [10]. These are cohort‐specific estimates rather than population‐wide prevalence rates, but together they support assessment for spinal infection when patients with IE report new focal back pain and consideration of a cardiac source when vertebral osteomyelitis occurs with bacteremia or predisposing valvular disease. In our patient, severe back pain and inability to walk led to the recognition of multilevel vertebral infection with epidural extension.

A second consideration is that vegetation size does not establish the extent of extracardiac disease. A meta‐analysis of 21 studies involving 6646 patients found that vegetations larger than 10 mm were associated with increased odds of embolic events, but a smaller vegetation does not exclude such complications [11]. The vegetation in our patient measured approximately 6 mm, whereas imaging also revealed extensive spinal infection and a 6.2‐cm splenic infarction. Measurements obtained at diagnosis cannot reconstruct vegetation size before a potential embolic event, and the patient′s atrial fibrillation provides an alternative possible explanation for the splenic infarction. Nevertheless, the clinical lesson remains: when symptoms suggest extracardiac involvement, a relatively small vegetation should not deter investigation.

A third consideration is the challenge of treatment selection in TAVR‐associated IE. Guidelines identify heart failure, uncontrolled infection, and prevention of embolization in selected high‐risk settings as major considerations for cardiac surgery [12, 13]. In an international registry of 584 patients with TAVR‐associated IE, surgical treatment was not associated with lower adjusted in‐hospital or 1‐year mortality than antibiotic treatment alone [14]. A subsequent meta‐analysis of three studies involving 1557 patients likewise found no statistically significant pooled difference in 1‐year survival, although between‐study heterogeneity was substantial [15]. These observational findings do not establish equivalence between the two approaches, and surgery remains important when appropriate indications are present. In our patient, extensive extracardiac disease coexisted with no identified perivalvular abscess or fistula and no documented hemodynamic instability. His age and comorbidities informed the decision to pursue antimicrobial treatment without valve surgery.

Antimicrobial therapy was guided by organism identification and susceptibility testing. Once *G. haemolysans* was identified, empiric vancomycin was discontinued and ceftriaxone was continued. The planned 6‐week course reflected the combination of prosthetic‐valve infection and vertebral osteomyelitis, for which prolonged treatment is generally appropriate [12, 13]. Fever resolved and inflammatory markers declined during hospitalization.

## 4. Conclusion

We report *G. haemolysans* prosthetic‐valve endocarditis following TAVR, accompanied by multilevel lumbar discitis and osteomyelitis, epidural abscess, a right psoas collection, and splenic infarction. Three lessons emerge: endocarditis and vertebral osteomyelitis merit reciprocal diagnostic consideration; vegetation size alone cannot define the burden of extracardiac complications; and the choice between antimicrobial and surgical management after TAVR requires assessment of specific indications and procedural risk.

## Funding

No funding was received for this manuscript.

## Disclosure

This research was supported in whole or in part by HCA Healthcare and/or an HCA Healthcare affiliated entity. The views expressed in this publication represent those of the authors and do not necessarily represent the official views of HCA Healthcare or any of its affiliated entities.

## Conflicts of Interest

The authors declare no conflicts of interest.

## Data Availability

The data that support the findings of this study are available from the corresponding author upon reasonable request.
